# Intrahepatic flow diversion prior to segmental Yttrium-90 radioembolization for challenging tumor vasculature

**DOI:** 10.1016/j.jimed.2022.02.001

**Published:** 2022-05-21

**Authors:** Lindsay B. Young, Marcin Kolber, Michael J. King, Mona Ranade, Vivian L. Bishay, Rahul S. Patel, Francis S. Nowakowski, Aaron M. Fischman, Robert A. Lookstein, Edward Kim

**Affiliations:** a Icahn School of Medicine at Mount Sinai Department of Diagnostic, Molecular and Interventional Radiology, One Gustave L. Levy Place Box 1234, NY, 10029-6574, New York, USA; b University of Texas Southwestern Medical Center, Division of Vascular and Interventional Radiology, 5323 Harry Hines Blvd, TX, 75390-9316, Dallas, USA; c David Geffen School of Medicine at the University of California – Los Angeles, Department of Interventional Radiology, 27235 Tourney Road, Suite 1500, California, 91355, Valencia, USA

## Abstract

**Background:**

Hepatic tumors with complex vascular supply or poor relative perfusion are prone to decreased rates of objective response. This is compounded in the setting of Yttrium-90 (Y90) transarterial radioembolization (TARE), which is minimally embolic and flow-dependent, relying on high threshold dose for complete response.

**Objective:**

We describe our experience with intrahepatic flow diversion (FD) prior to TARE of hepatocellular carcinoma (HCC) with challenging vascular supply.

**Materials and methods:**

Between April 2014 and January 2020, 886 cases of coinciding MAA or TARE and bland embolization or temporary occlusion were identified. Intraprocedural embolizations performed for more routine purposes were excluded. FD was performed by bland embolization or temporary occlusion of vessels supplying non-malignant parenchyma in cases where flow was not preferential to target tumor. Lesion characteristics, vascular supply, treatment approach, angiography, and adverse events (AEs) were reviewed. Radiographic response was assessed using mRECIST criteria.

**Results:**

22 cases of FD of focal HCC were identified. Embolics included calibrated microspheres (n ​= ​11), microcoils (n ​= ​4), gelfoam (n ​= ​3), temporary balloon occlusion (n ​= ​2) and temporary deployment of a microvascular plug (n ​= ​1). Post-treatment SPECT-CT dosimetry coverage was concordant with target lesions in all cases. Mean follow-up was 16.7 months (1.4–45 ​mos). Tumor-specific response per mRECIST was 41% complete response, 50% objective response, and 59% disease control rate. No major adverse events or grade 3/4 hepatotoxicity were reported.

**Conclusion:**

Our findings suggest that FD prior to TARE is safe and potentially effective in treating HCC with complex vascular supply or poor tumor perfusion.

## 1 Introduction

Yttrium-90 (Y90) transarterial radioembolization (TARE) is a well-established therapy for treatment of hepatocellular carcinoma (HCC), demonstrating high rates of complete response in selective administration.^1^^,^^2^ The dominant vascular supply of HCC is via the hepatic arteries, as opposed to the normal liver parenchyma, which receives most of its blood supply from the portal veins. This feature allows for selective treatment of tumor via the transarterial route while sparing normal hepatic parenchyma. However, tumors with complex vascular supply including multiple or inaccessible feeding vessels, and/or poor tumor perfusion are prone to decreased rates of objective response.^3^ As TARE is a minimally embolic, flow-dependent therapy that utilizes radiation dose threshold for response, the aforementioned obstacles present a challenge to obtaining complete response in the target tissue. Furthermore, cases of HCC with complex vascular supply may be predisposed to non-target embolization of non-tumoral parenchyma during TARE.^4^^,^^5^ A technique of intrahepatic flow diversion prior to TARE has been described in a limited number of patients, most recently by Core et al., with the goal to improve delivery of Y90 and decrease dose to tumor-adjacent normal liver parenchyma.^6^ We report on our institutional experience of the feasibility, safety and efficacy of this technique.

## 2 Materials and Methods

**Fig. 1:**
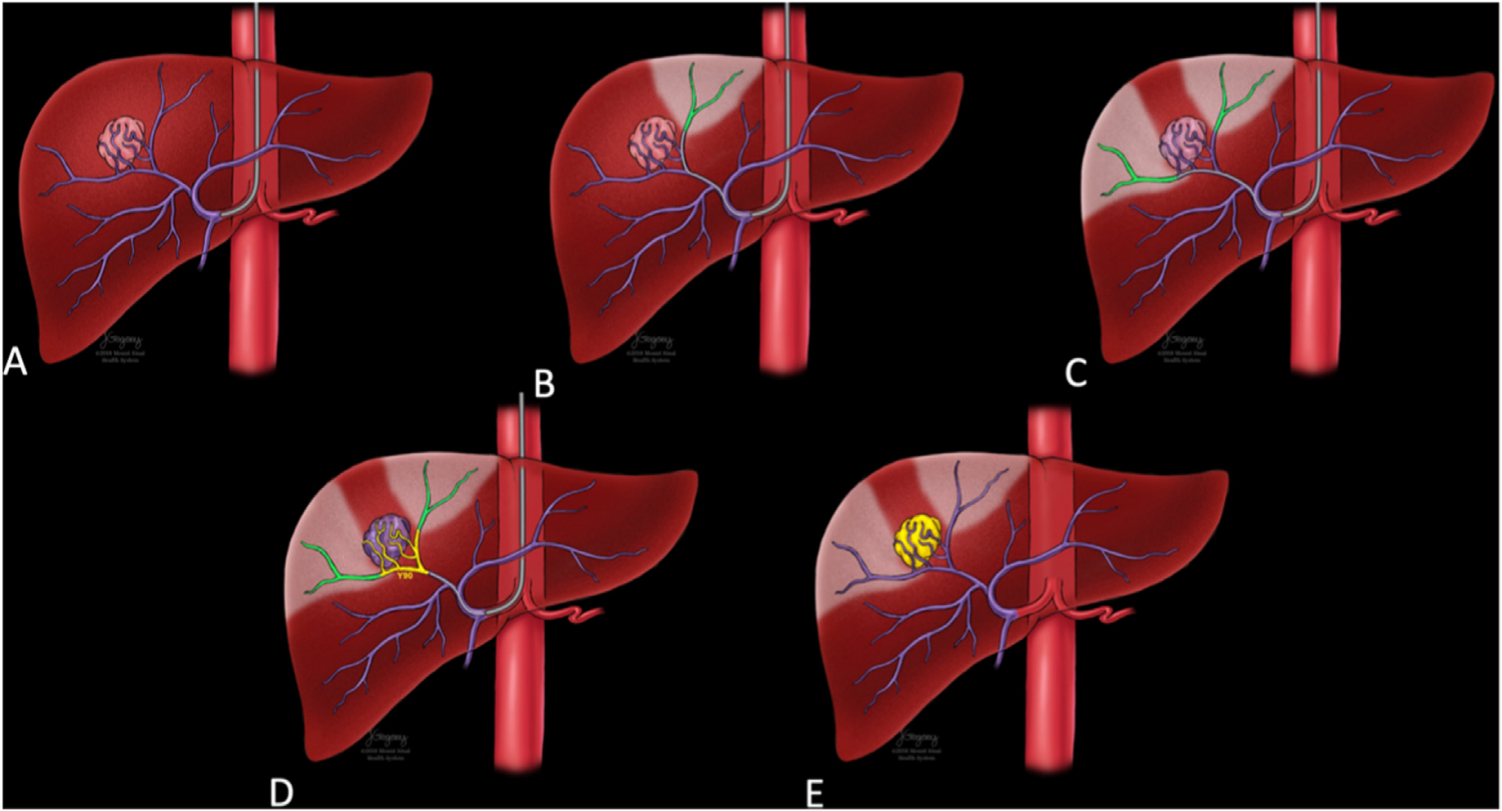
Technique of Flow Diversion Prior to Y90 Transarterial Radioembolization. Temporary or permanent occlusion of vessel(s) (green vessels) supplying normal liver parenchyma that are part of a competing vascular bed to the tumor (B, C). Flow is augmented to the tumor prior to delivery of Y90 (yellow vessels) with additional distal protection of the surrounding normal liver parenchyma provided by the adjacent arterial occlusion (green vessels) (D, E).

**Fig. 2:**
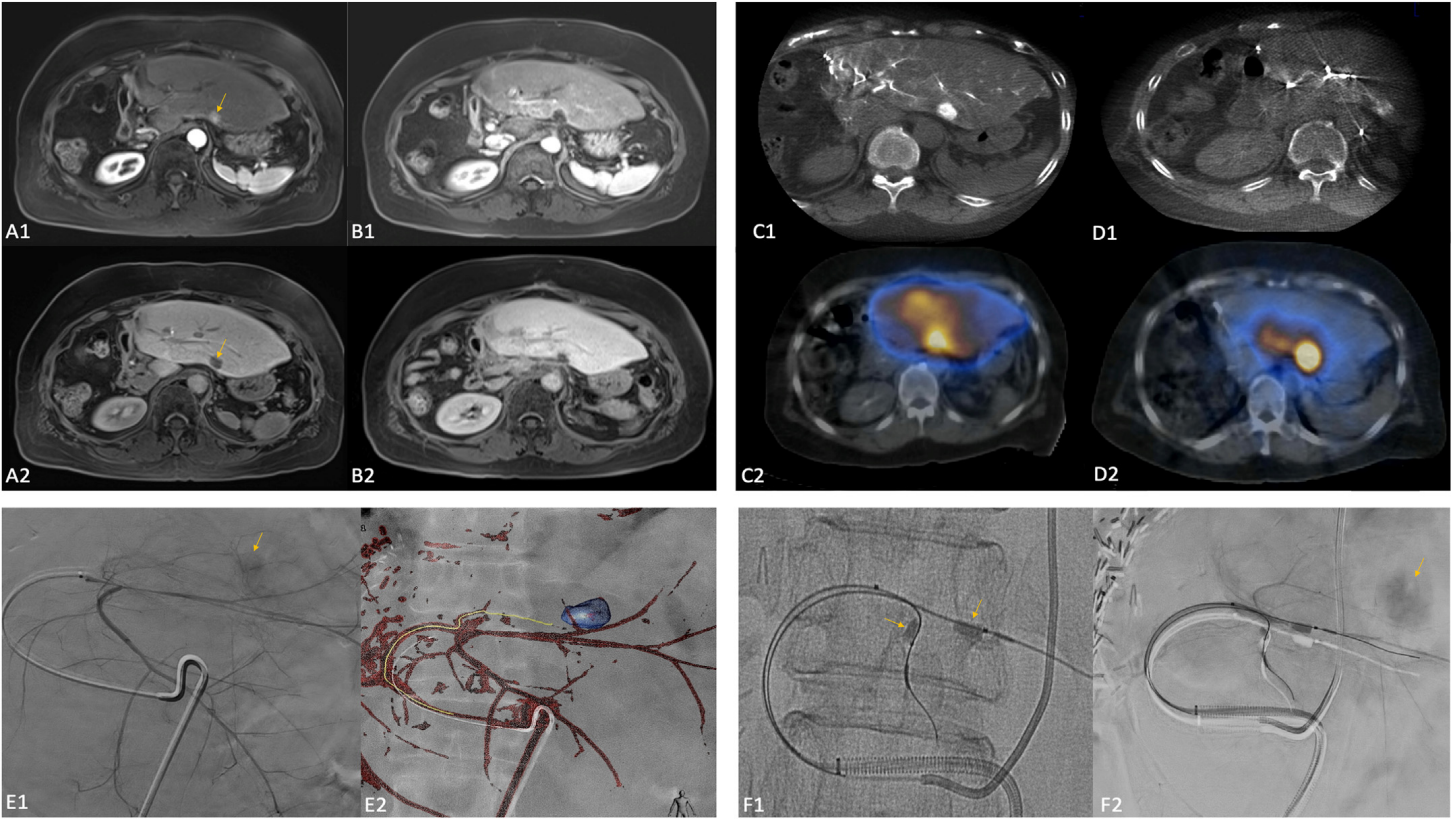
Case 1.68 year old female patient with HCC status post right hepatectomy with tumor recurrence in the left hepatic lobe as demonstrated on arterial phase and hepatobiliary phase MRI (A1 and A2, arrows). Left hepatic angiography (E1 and E2) as well as CBCT and SPECT-CT (C1 and C2) from the MAA mapping procedure demonstrated enhancement/uptake of the target segment (arrow) as well as the surrounding parenchyma. CBCT and SPECT-CT after balloon occlusion of the distal segment 2 and 3 arterial branches (F1 and F2, arrows) demonstrated augmented flow to the tumor (arrow) and decreased flow to the surrounding parenchyma. Follow-up arterial phase and hepatobiliary phase MRI one year post-treatment (B1 and B2) demonstrated complete response and minimal radiation effect to the surrounding parenchyma.

A retrospective review was performed of patients who underwent flow diversion prior to TARE during either macroaggregated albumin (MAA) mapping or Y90 radioembolization procedures. This study was approved by our institutional review board. All clinical practices and observations were conducted in accordance with the Declaration of Helsinki. Informed consent was waived because of the retrospective nature of the study and the analysis used anonymous clinical data. All cases of flow diversion were performed during macro-aggregated albumin (MAA) mapping procedure or Y90 TARE. Procedures were reviewed from January 2014 through January 2020, yielding 886 cases. All cases of concomitant bland embolization determined to represent flow diversion were included in this study. Flow diversion was defined as the occlusion of vessels supplying non-malignant hepatic parenchyma with the goal of enhancing arterial flow into targeted tumor(s). Procedures were performed by interventional radiologists ranging from four to greater than 20 years of experience. Decision to perform flow diversion was at the discretion of the interventional radiologist in cases where highly selective Y-90 delivery could not be performed, and a large volume unaffected liver would otherwise be exposed to radioembolization from a proximal delivery site (Fig. 1, Fig. 2). In these cases, it was believed that distal bland embolization would both protect non-malignant liver from therapeutic radiation while improving flow to vessels supplying tumor. Embolization performed for more routine purposes such as extrahepatic protective embolizations (i.e. gastroduodenal, gastric, cystic, or retroportal arteries), flow redistribution from extrahepatic feeding vessels (i.e. inferior phrenic arteries), as well as cases of arterioportal shunt embolization were excluded. In total, 22 cases of flow diversion were identified.

Prior to treatment, all patients had previously undergone a standardized pre-treatment work-up including clinical, imaging and laboratory evaluation. A Medical Internal Radiation Dose Committee (MIRD) was used for dosimetry of Y90 glass microsphere treatments. Choice of embolic for flow diversion was at the operator's discretion, with microspheres, microcoils, gelfoam, temporary balloon occlusion or temporary deployment of a microvascular plug (MVP) for the purposes of enhancing flow to the tumor and preventing nontarget embolization of normal tissue.

Planar and tomographic images were acquired by single-photon emission computed tomography (SPECT-CT) after MAA mapping and Bremsstrahlung imaging post-Y90. These studies were compared to pre-procedure MRI and, when applicable, intra-procedural cone-beam CT (CBCT) to evaluate dose delivery distribution. Post-procedure SPECT-CT or Bremsstrahlung scans were reviewed by two independent radiologists to confirm coverage of tumor by radiopharmaceutical (MAA) or radioembolic (Y90).

All patients were discharged home the same day of the procedure with oral analgesic and antiemetic agents as needed, as well as a 7-day course of levofloxacin.

Primary outcome was technical procedural success as outlined by the SIR quality improvement guidelines for hepatic malignancies, defined as successful administration of Y90 microspheres to the target tumor tissue during the Y90 administration component of the procedure as reflected in post-Y90 Bremsstrahlung scan. Secondary outcomes were tumor response and safety. Modified Response Evaluation Criteria In Solid Tumors (mRECIST) were used to grade imaging response at six weeks and beyond, and was reported at the longest existing imaging follow-up time point at which the tumor had not been retreated or resected, or the patient had not undergone liver transplantation. mRECIST score was assigned after review of pre- and post-treatment imaging by an independent radiologist, specializing in body MRI, with three years of experience. Patients with multifocal and infiltrative HCC were excluded from the study due to difficulty in attributing flow diversion to a specific lesion or intervention. Clinician- or patient-reported adverse events, as well as liver function tests were reviewed up to three months post-procedure to identify subclinical or sustained hepatotoxicity. These were reported in accordance with Version 5.0 of the Common Terminology Criteria for Adverse Events.^7^

## 3 Results

Flow diversion was performed during the mapping (n ​= ​6) or Y90 (n ​= ​16) component of the procedure. All TARE was performed using Y90 glass microspheres (Therasphere, BTG, UK). Median tumor diameter was 3.1 ​cm (range 1.3–6.5 ​cm). Intraprocedural CBCT was performed in 12/22 cases (52%). Bland embolization of hepatic tissue was performed in the lobe ipsilateral to the target tumor in all cases (right lobe n ​= ​15, left lobe n ​= ​7). Embolics used included microspheres (size range 100–500 ​μm; 11/22 cases, 50%), microcoils (size range 2–4 ​mm; 4/22 cases, 18%), gelfoam (3/22 cases, 14%), temporary balloon occlusion (3/22 cases, 14%), and temporary deployment of a microvascular plug (MVP, Medtronic) which was re-sheathed and retrieved at the completion of Y90 administration (1/22 cases, 5%).

In 11/22 cases (50%), non-tumor-supplying branches distal to the tumor-supplying branches within the same hepatic segment were occluded to divert flow proximally towards the tumor. In the remaining cases, flow was diverted from a different, competing segment to the one containing tumor. In all cases, SPECT-CT or Brehmsstrahlung scans demonstrated uptake commensurate with tumor location or post-procedure imaging.

The mean follow-up time was 16.6 months (range 1.4–45 months). There were no major adverse events or cases of grade 3/4 hepatoxicity. Minor complications included one case of grade I hepatotoxicity, one grade II hematoma and one instance of post-procedural pain attributed to post-embolization syndrome. Per mRECIST criteria, complete response (CR) rate was 41%, objective response rate was 50%, and the disease control rate was 59%. Patient, treatment, follow-up, and response characteristics are described in Table 1.

**Table 1:** Patient, tumor, treatment and response characteristics.

	Childs-Pugh	Tumor Diameter (cm)	BCLC Stage	Embolic type	Vessel/Segment embolized for flow diversion	Vessel/segment treated	Complications	Follow-up duration	mRECIST
1	A	6.5	B	Particle	II/III	IV	–	5	SD
2	A	2.6	B	Particle	VI	VI	–	12	CR
3	A	4.8	A	Particle	V/VIII	V	Grade II groin hematoma	3	PD
4	A	3.3	A	Particle	VI/VII	VI	Pain	18	PD
5	A	3	A	Particle	VII	VI/VII	–	28	CR
6	A	4.1	B	Particle	V/VII	I/VIII	–	25	PD
7	B	2.6	B	Coil	VIII	V	Grade 1 hepatotoxicity	2.5	PD
8	B	4.6	C	Coil	VIII	VIII	–	4	PR
9	A	3	A	Coil	IV	II/III	–	15.5	CR
10	A	2.6	A	Particle	VIII	VIII	–	39	PD
11	A	1.6	A	Gelfoam	V	V	–	24	SD
12	A	2.5	B	Particle	V/VI	V/VI	–	1.4	PR
13	A	1.6	A	Particle	III	II/III	–	45	CR
14	A	2	A	Particle	VII	VI	–	44	CR
15	A	1.6	B	Coil	VI	VII	–	18.5	PD
16	A	1.6	A	Temporary Microvascular Plug Occlusion	VII	VI/VIII	–	5	CR
17	A	5.7	A	Gelfoam	II/III	Common IV/Left hepatic artery trunk	–	12.5	PD
18	A	2.2	A	Gelfoam	V branch	VIII	–	5.5	CR
19	A	1.3	A	Particles	IV branch	IV branch	–	34	CR
20	A	6.2	A	Temporary Balloon Occlusion	IVa	IV	–	4.5	PD
21	A	1.7	A	Temporary Balloon Occlusion	III	II	–	1.5	PD
22	A	1.7	A	Temporary Balloon Occlusion	II/III	II	–	18.5	CR

MVP: Microvascular Plug; BCLC: Barcelona-Clinic Liver Cancer; “-“: No complication; mRECIST: Modified Response Evaluation Criteria in Solid Tumors; CR: Complete Response; PR: Partial Response; SD: Stable Disease; PD: Progression of Disease.

∗MVP was re-sheathed and removed following Y90 administration).

## 4 Discussion

The present study illustrates the safety and potential efficacy of altering intrahepatic arterial flow to improve Y90 delivery to target tumor in cases of TARE that would have otherwise been technically difficult or prone to treatment failure due to complex anatomy. We found moderate overall rates of objective response with no major adverse events reported.

Tumors best suited to flow diversion are central lesions that demonstrate multiple arterial feeders arising from the proximal portions of segmental arteries. These vessels are not amenable to individual catheterization due to their small size or sheer number. In some cases, these lesions are only minimally hypervascular relative to the surrounding liver parenchyma due to competitive flow to the larger distal arteries, which is further problematic for flow-directed therapy. Because of these features, proximal delivery of Y90 would tend to incompletely treat these tumors. The occlusion of distal and/or competing parenchymal bed(s) appears to alter and preferentially increaseflow into the tumor, increasing the deposition of Y90 to target tissue.

Cases in which a tumor is partially supplied by parasitized arteries may benefit from flow redistribution (FR).^8^ In FR, the nontarget vessel is embolized, usually with coils and/or microparticles during the mapping component of the procedure, with the expectation that this fraction of tumor will regain supply from the dominant feeder through which Y90 will be subsequently delivered. This is also referred to as “arterial consolidation” and has been used in a variety of treatment scenarios including the presence of extrahepatic feeding vessels, cases of multiple intrahepatic feeding vessels that are not each amenable to Y90 administration or to simplify Y90 dosage calculation and administration by treatment through a single vessel.^9^, ^10^, ^11^, ^12^

It is valuable to distinguish ‘flow redistribution’ and ‘flow diversion’. While independently useful in different circumstances, the techniques are opposite in nature: flow redistribution involves embolization of competing tumor vascular supply extrinsic to the site of Y90 delivery in order to encourage perfusion of target tissue to intrahepatic collaterals arising from the Y90 treatment bed, either via vasodilation of dormant collaterals or through neo-angiogenesis. In contrast, flow diversion utilizes blockade of adjacent healthy liver parenchyma in order to both protect this tissue from Y90 deposition and redirect flow into the tumor, without relying on functional changes to the tumor's vascular supply.

This technique was described recently by Core et al. in a subset of patients with different types of hepatic tumors, demonstrating significant dose reduction to tumor-adjacent parenchyma upon analysis of SPECT-CT images.^6^ A variation of a flow diversion technique was first described by Itagaki et al. in two cases of transarterial chemoembolization (TACE) for which vessels supplying tumor were difficult to catheterize individually.^3^ Rather than embolizing proximally and risking substantial nontarget deposition of chemoembolic to the entire right lobe, the author advanced a balloon microcatheter distal to the feeding vessels, temporarily inflated the balloon, and delivered the dose through the guiding catheter (around the microcatheter) from the proper hepatic artery.

Another distal occlusion technique was described in a small case series by Meyer et al., who successfully utilized degradable starch microspheres to protectively embolize healthy liver parenchyma in a temporary fashion immediately prior to TARE in cases of complex vascular supply.^13^ Flow diversion is useful for TARE, as a modality, because the dominant treatment effect is from threshold radiation dose rather than ischemia. Y90 glass microspheres are minimally embolic and are dependent on preferential flow to distribute throughout the volume of tissue injected with the distribution closely approximating contrast density, especially at low injection rates.^13^^,^^14^ TACE, in contrast to TARE, elicits real-time changes in flow distribution attributed to gradual vessel occlusion, ensuring treatment of the entire parenchymal bed, especially with embolization to stasis or near-stasis. The sequential occlusion of vessels causes flow to redirect to patent vessels. We therefore refer to TARE as a “flow-dependent” therapy. Flow diversion, in theory, assures that the target tumor receives the majority blood flow, and therefore majority of radiation dose, especially in cases of decreased intrinsic vascularity.

In our experience, flow diversion did not result in substantial hepatotoxicity or large volume tissue necrosis. Only one patient experienced pain attributable to a post-embolization syndrome. One cases of grade I hepatotoxicity was identified. There was no grade III or IV hepatotoxicity. It should be noted that no patient had portal vein occlusion, and bland microsphere embolization was not performed to stasis or even near stasis: the endpoint was mild pruning of distal vessels and care was taken to avoid reflux into tumor feeders. Embolics below 100 ​μm in diameter were not used. The risk of premature occlusion of vessels supplying tumor led some operators to opt for microcoil embolization instead. Temporary occlusion, such as with a microvascular plug or balloon occlusion catheter, was found to be effective and operator dependent. However, this technique required either upsizing to a 6 French guide sheath to accommodate two microcatheters, or two access points. Gelfoam is an attractive alternative due to its temporary nature but the inconsistent particle size and injection rates associated with a slurry injection raise concerns for reflux into tumor vasculature.^15^

It is unclear how long the embolic effect of sub-therapeutic bland embolization of normal liver actually lasts. The regenerative capabilities of the liver suggest that neovascularity and collateral formation post-embolization occursand thus it is unclear if the one to two week time period between mapping and Y90 radioembolization is short enough to sustain the effects of flow diversion.^9^^,^^16^^,^^17^ As a result, flow diversion may benefit from being utilized during the Y90 delivery, to maximize the benefit of distal vessel occlusion and minimize recanalization of flow to embolized branches.

Limitations of the study include its retrospective nature and small sample size. Additionally, the iterative nature of employing this technique early on resulted in significant variability in embolization technique between operators and patients. Our institutional technique has become more uniform and protocoled over time, with nearly all cases of intrahepatic FD now being performed during the radioembolization procedure with temporary balloon occlusion, immediately prior to Y90 delivery. However, we believe the efficacy of each flow diversion embolization technique to be comparable for the purposes of this study.

In summary, our findings suggest that flow diversion is a technically feasible, safe and potentially effective technique to augment Y90 delivery to tumor and protect normal liver parenchyma in the setting of tumors that may not be amenable to successful treatment with proximal delivery of TARE. Further investigation comparing TARE in the presence and absence of flow diversion in tumors of similar characteristics is warranted to evaluate the comparative safety and efficacy of this technique.
